# Endometrial stem cell transplantation in MPTP- exposed primates: an alternative cell source for treatment of Parkinson's disease

**DOI:** 10.1111/jcmm.12433

**Published:** 2014-10-06

**Authors:** Erin F Wolff, Levent Mutlu, Efi E Massasa, John D Elsworth, D Eugene Redmond, Hugh S Taylor

**Affiliations:** a Department of Obstetrics, Gynecology and Reproductive Sciences, Yale School of MedicineNew Haven, CT, USA; b Department of Psychiatry, Yale School of MedicineNew Haven, CT, USA; c Department of Neurosurgery, Yale School of MedicineNew Haven, CT, USA; d Department of Molecular, Cellular and Developmental Biology, Yale UniversityNew Haven, CT, USA

**Keywords:** adult stem cells, endometrium, mesenchymal stem cells, MPTP, neurodegenerative diseases, Parkinson disease, stem cells, transplantation

## Abstract

Parkinson's disease (PD) is a neurodegenerative disease caused by the loss of dopaminergic neurons in the substantia nigra. Cell-replacement therapies have emerged as a promising strategy to slow down or replace neuronal loss. Compared to other stem cell types, endometrium-derived stem cells (EDSCs) are an attractive source of stem cells for cellular therapies because of their ease of collection and vast differentiation potential. Here we demonstrate that endometrium-derived stem cells may be transplanted into an MPTP exposed monkey model of PD. After injection into the striatum, endometrium-derived stem cells engrafted, exhibited neuron-like morphology, expressed tyrosine hydroxylase (TH) and increased the numbers of TH positive cells on the transplanted side and dopamine metabolite concentrations *in vivo*. Our results suggest that endometrium-derived stem cells may provide a therapeutic benefit in the primate model of PD and may be used in stem cell based therapies.

## Introduction

Parkinson's disease (PD) is a common neurodegenerative disease, affecting 1–2% of the population over the age of 55 [1]. It is characterized by progressive loss of dopaminergic neurons of the substantia nigra resulting in insufficient dopamine (DA) concentrations within the striatum. Progressive loss of these neurons ultimately causes debilitating motor, behavioural, sensory and autonomic symptoms. Although a wide variety of drugs, ablative surgeries and deep brain stimulation treatments are used to ameliorate the symptoms, these therapies have not been able to replace the lost cells or effectively slow down the relentless neurodegenerative process. Therefore, interest in cell-replacement therapies has greatly increased, ultimately aiming to supplant the lost dopaminergic neurons in the substantia nigra with healthy dopamine-producing neurons, and to protect the endogenous neurons from further loss [2]. Many experimental models of PD have been developed to study the efficiency and feasibility of cell-replacement therapies in animals. Many of those models utilize 1-methyl-4 phenyl-1,2,3,6-tetrahydropyridine (MPTP) to induce selective neurotoxicity of the nigral dopaminergic neurons and parkinsonism in the recipient animals. Subsequent transplantation of foetal mesencephalic tissue, embryonic stem cells (ESC) and induced pluripotent stem cells (iPS) have resulted in varying degrees of success in replacing DA neurons. However, ethical considerations, difficulties in finding a continuous supply of foetal mesencephalic tissues and ESCs, and the risk of tumour formation after ESC or iPS transplant that have been reprogrammed to a pluripotent state, all hamper their potential clinical application. However, mesenchymal stem cells represent a promising tool for cell-replacement therapy in PD. Previous research has shown their ability to promote endogenous neural growth, decrease apoptosis, reduce levels of free radicals in the microenvironment, induce synaptic formation, and regulate inflammation [3–5].

The human endometrium contains a rich supply of mesenchymal stem cell-like cell population with remarkable differentiation capacity [6]. Moreover, the endometrium is the only source of tissue derived mesenchymal stem cells (EDSCs) that can be obtained without analgesic requirements, thereby rendering these cells as a feasible source for stem cell therapies. Previously, our group showed the ability of human EDSCs expressing CD90, PDGF-Rβ and CD146 to differentiate into dopamine-producing neuron-like cells *in vitro* with a two-step differentiation protocol. Differentiated cells demonstrated dendritic-like and axon-like projections that recapitulate synapse formation. These cells in addition, expressed neural cell markers nestin and tyrosine hydroxylase, which is the rate-limiting enzyme in dopamine synthesis. Whole-cell patch clamp recording of the differentiated cells verified the presence of inwardly rectifying Barium-sensitive G-protein coupled potassium channels (GIRK2), which are characteristic of the central neurons within the substantia nigra including the dopaminergic cells. Moreover, transplantation of undifferentiated EDSCs into the striatum of MPTP-treated mice showed that the transplanted cells can engraft into the striatum, migrate to the substantia nigra, spontaneously differentiate *in vivo* and increase striatal dopamine and dopamine metabolite concentrations [7]. We sought to investigate their potential for clinical cellular therapies using a non-human primate model in a pilot feasibility study. Here, we examine the potential of EDSCs for transplantation for the first time in non-human primates using a dopamine depletion model of PD.

## Materials and methods

### Animals

Eight adult female and eight adult male St. Kitts green monkeys (*Chlorocebus sabaeus*) were used in accordance with the standards and approval of the institutional animal care and use committee of Axion Research Foundation. Animals were trapped and kept in individual cages to facilitate any special care and feeding requirements. They were fed standard amounts of Harlan Teklad monkey chow per body-weight and had free access to water. Female monkeys served as endometrial tissue donors. As previously described in detail by Brunet *et al*., three male monkeys exposed to MPTP served as tissue recipients [8]. Five age-matched male monkeys did not receive MPTP treatment and served as controls to determine the normal homovanillic acid (HVA) concentrations in St.Kitts green monkeys.

### MPTP treatment

To deplete dopamine-producing neurons, three adult male monkeys received standard low-dose (0.4 mg/kg/12 hrs times three doses to reach the total dose of 1.2 mg/kg) MPTP as previously described [8–12].

In this pilot study, low-dose MPTP was preferred over higher doses to determine the biological effects of endometrium-derived stem cell transplantation before subjecting monkeys to greater Parkinsonian deficits and difficulties caring for themselves. Based on previous studies using the same species and dose, we estimated that the dose we used would reduce dopamine levels by 50–80%, however, the monkeys would be essentially asymptomatic [8].

### Endometrial sample collection

Endometrial tissue was collected by hysterectomy and curettage from eight monkeys. Standard endometrial stromal cell cultures were generated in a routine fashion, which produced an unfractionated stromal cell population. Briefly, endometrial tissue was minced and then digested in HBSS (Gibco, Invitrogen, Carlsbad, CA, USA) containing HEPES (25 mM), Collagenase B (1 mg/ml, Roche Diagnostics, Indianapolis, IN, USA) and DNase I (0.1 mg/ml, Sigma-Aldrich, St. Louis, MO, USA) for 30–45 min. at 37°C with agitation. Resultant dispersed cell solutions were then passed through a 70 μm sieve (BD Biosciences, Beford, MA, USA) to remove glandular epithelial components. Filtered cell solutions were centrifuged, and the supernatant was decanted and resuspended in minimal essential media (Gibco, Invitrogen) with phenol red, 1% antibiotics-antimycotics (Gibco, Invitrogen), and 20% foetal bovine serum (Gibco, Invitrogen). Resuspended cells were then plated in plastic flasks and maintained at 37°C in a humidified chamber (5% CO_2_) for expansion for 1 week. Culture medium was changed every other day. We have previously shown that this technique enriches for EDSCs from fresh endometrial samples. EDSCs display strong positivity for MSC markers such as PDGF-Rβ (99.7%), CD146 (99.7%) and CD90 (99.6%) [7].

### Transplantation of endometrium-derived stem cells to the MPTP-treated primates

Endometrial cells were trypsinized and stained with PKH26 (Sigma-Aldrich) according to the manufacturer's recommendations. PKH26 is a fluorescent dye that stably incorporates into the lipid regions of the cell membrane. The cells were resuspended in 40 μl of HBSS, thereby making a concentration of 100,000 cells/μl for the injection.

All three MPTP-treated male primates were deeply anesthetized with intravenous pentobarbital under sterile surgical conditions. Injections were made with a 22 gauge needle attached to a 100 μl Hamilton syringe driven by a Stoelting microinjector at a rate of 2 μl/min. The needles were withdrawn 2 min after injection, at a rate of 1 mm/min for the initial 5 mm, and then slowly thereafter to avoid suctioning cells into the injection track. Two sites in the right caudate nucleus were injected with 40 μl of PKH26 stained endometrium-derived cells. Only the right caudate nuclei were injected in the recipient primates. The left caudate nucleus in each monkey was injected with an equal volume of culture medium, thereby serving as internal controls.

The stereotaxic targets in the right caudate nuclei were AP+23.1 mm and 19.1 mm, lateral 4 mm on each side from the midline, +19 mm above earbar [13].

### Necropsy

One month after injection all three monkeys were killed using ketamine (8–10 mg/kg IM) and pentobarbital (30 mg/kg IV or more until loss of corneal reflex). The monkey brains were perfused with heparinized saline and fixed with 4% paraformaldehyde in phosphate buffer before being cryopreserved in 30% sucrose solution. Sections (50 μm thick) were prepared with a cryotome and were stored at −80°C until later use in 50 mM phosphate buffer (pH 7.4) with 25% glycerol cryoprotection solution.

### Immunofluorescence and cell counts

Sections were permeabilized and blocked in PBS containing 0.2% Triton (Promega, Madison, WI, USA), 10% normal goat serum (Jackson ImmunoResearch, West Grove, PA, USA) and 1% bovine serum albumin (Sigma-Aldrich) for one hour before being incubated overnight at 4°C with a mouse polyclonal anti-tyrosine hydroxylase antibody (1:5000; EMD Milipore AB152, Santa Cruz, CA, USA) followed by Goat antimouse Alexa Fluor 594 (1:1000, Invitrogen Probes, Invitrogen, Eugene, OR, USA) secondary antibody for one hour at 37°C. Slides were mounted using medium with ProLong® Gold antifade reagent (Invitrogen, Paisley, PA4 9RF, UK). All the samples were immediately viewed using a Carl Zeiss LSM 710 Duo Confocal microscope with appropriate filters to detect Alexa Fluor 594 and PKH26. Images were analysed using Zen Software (software available at: http://microscopy.zeiss.com). The number of tyrosine hydroxylase positive cells was counted in multiple low-power fields on each of four slides from three animals and averaged for each experimental animal. The percentages of the EDSC-derived cells that migrated to the substantia nigra and of the EDSC-derived TH(+) cells were estimated based on the average number of EDSCs in the total volume of tissue sections that were used for immunofluorescence. The formula used to estimate the cell counts was modified from Theoret *et al*. [14]: *N* = V_(SN)_ (∑Q/∑V_(SEC)_), where *N* is the total number of cells, V_(SN)_ is the total volume of substantia nigra, ∑Q is the total number of EDSCs counted in the low-power field immunofluorescence images and ∑V_(SEC)_ is the total volume of tissue sections that were analysed in low-power field images.

### HVA concentrations in striatum

To test the effect of EDSC transplantation on the striatal dopamine levels, we have measured the principal metabolite of dopamine, HVA concentration on both sides of the brain using HPLC as previously described [15]. DA values were not available for these samples because of a technical difficulty. However, HVA concentrations were measured, and we have shown that these levels reflect restoration of dopaminergic tone in the striatum of MPTP-treated monkeys following grafts of dopamine neurons [16]. It should be noted though that graft-induced increases in HVA levels are always of less magnitude than dopamine levels, so in the present study the change in dopamine levels would likely be greater than that reported for HVA.

### Statistical analysis

Cell counts on the transplanted and non-transplanted sides respectively were compared using Wilcoxon Rank Sum Test analysis using spss Statistical Software SPSS Inc. Released 2009. PASW Statistics for Windows, Version 18.0. Chicago: SPSS Inc. USA. The HVA levels and significance of the differences in the HVA levels in the striatum were analysed using *t*-test analysis. A one-sided *P* < 0.05 was considered statistically significant (Prism Software, La Jolla, CA, USA).

## Results

**Fig. 1:**
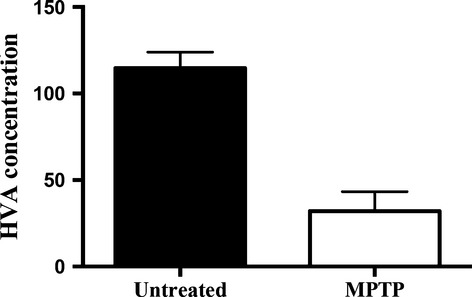
MPTP treatment resulted in a significantly lower HVA concentration in the recipient animals compared to the untreated animals (*P* < 0.05, *t*-test). Striatal HVA concentrations were measured in untreated monkeys and the sham-injected side of the MPTP-treated animals (MPTP)

As a stem cell source, endometrial samples were collected from eight monkeys at variable menstrual cycle phases. After 1 week of culture, endometrial cells from three monkeys proliferated well and reached sufficient stem cell counts for transplantation. MPTP was used to create experimental PD in males. Low-dose MPTP treatment was well tolerated by the animals. Throughout the experimental protocol, the MPTP-treated monkeys did not show Parkinsonian disabilities and maintained their asymptomatic status. Endometrium-derived stem cell transplantation was well tolerated by the recipient monkeys without any clinically significant complications. MPTP treatment caused approximately a 70% decrease in HVA concentration in the sham-transplanted side of the recipient monkeys compared with the control monkeys that did not receive MPTP (32 *versus* 114 ng/mg protein respectively, *P* < 0.05, *t*-test; Fig. 1).

**Fig. 2:**
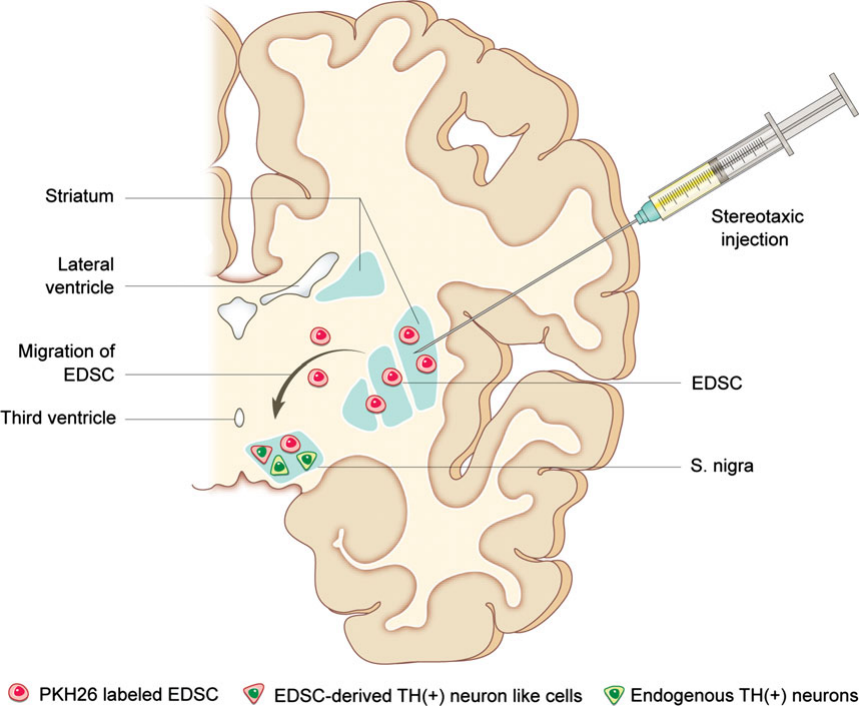
Schematic illustration of engraftment and migration of EDSCs. After stereotaxic injection, endometrium-derived stem cells (EDSCs) engrafted into the striatum and migrated to the substantia nigra, where some of the EDSCs exhibited neuron-like morphology and expressed tyrosine hydroxylase (TH).

PKH26 labelled endometrial stem cells were injected into the striatum; labelled cells were detected at the site of injection and were primarily distributed within the striatum. The transplanted cells were detected in the substantia nigra, which is ∼8 mm away from the striatal stereotaxic injection sites (Fig. 2). This observation suggests that EDSCs showed the ability to migrate from the striatum and engraft in the substantia nigra, which is consistent with published data on the migratory pattern of human neural stem cells implanted into the same locations [13]. The majority of cells remained in the striatum and the number reaching the substantia nigra was small (∼0.1%).

**Fig. 3:**
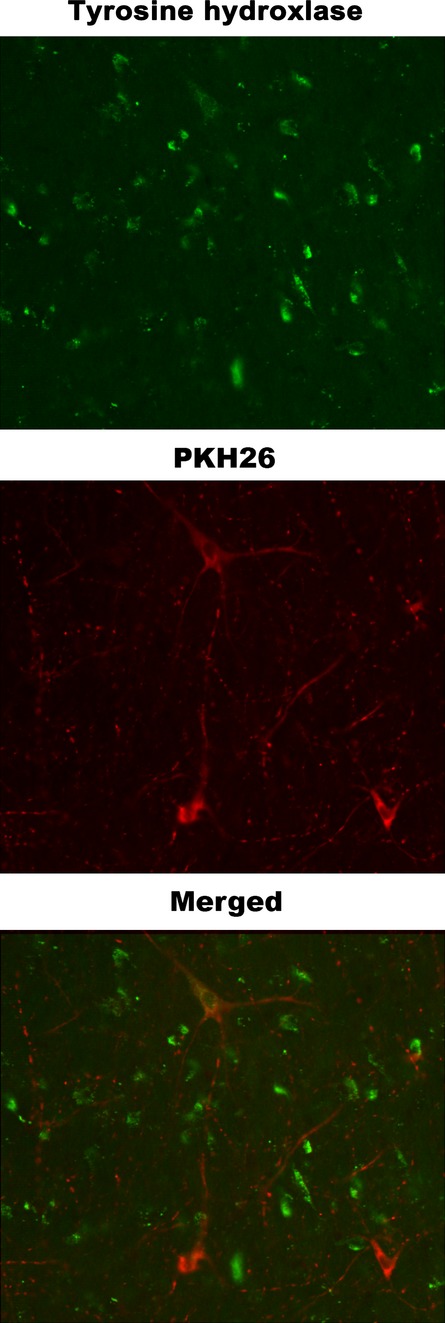
Tyrosine hydroxylase-expressing neuron-like cells derived from endometrial stem cells. Immunofluorescence analysis of PKH26-labelled EDSCs. Some of the PKH26- labelled EDSCs (red) exhibited neurite-like projections and expressed tyrosine hydroxylase (green) 4 weeks after intrastriatal injection (400×).

Approximately 0.01% of the transplanted cells differentiated from an endometrial phenotype into a neurogenic phenotype, as demonstrated by the development of neurite-like projections and tyrosine hydroxylase expression (Fig. 3). EDSC-derived TH(+) cells were ∼1% of all TH(+) cells observed. Transplanted cells did not show significant migration to the contralateral side. Although not statistically significant, the transplantation of endometrium-derived stem cells resulted in consistently higher numbers of TH(+) cells (stem cell-derived and endogenous) in the transplanted side of all animals (42 *versus* 62 on average in low-power field, Wilcoxon Rank Sum Test *P* = 0.10; Table 1).

**Table 1:** Average Tyrosine Hydroxylase (+) Cell Counts on the sham-injected side and the EDSC injected side of the brain in MPTP treated monkeys. Although not statistically significant, EDSC injection was associated with consistently higher cell counts on the recipient side of the brain. (Wilcoxon Rank Sum Test, *P* = 0.10)

	Control side	Transplant side
Monkey # 1	30	36
Monkey # 2	47	63
Monkey # 3	46	86

**Fig. 4:**
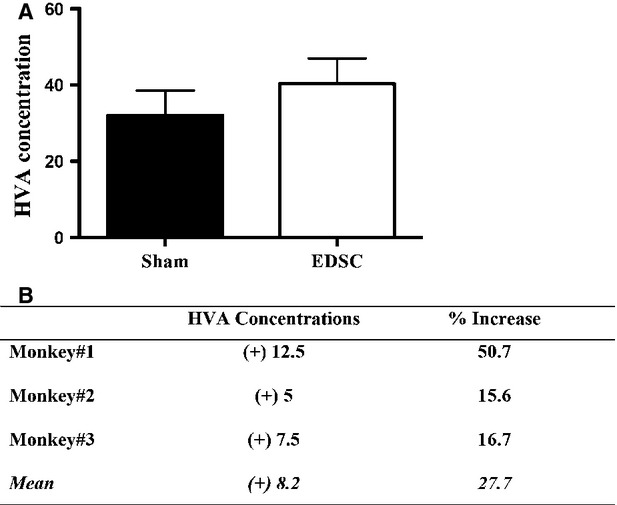
Striatal HVA Concentrations in MPTP-treated monkeys (ng/dl). (**A**) EDSC injection was associated with higher striatal HVA concentration compared with the sham injection. (**B**) Striatal HVA concentrations increased 12.5 ng/mg in Monkey #1, 5 ng/mg in Monkey #2 and 7.5 ng/mg in Monkey #3, with a mean increase of 8.2 ng/mg protein (*P* < 0.05, *t*-test) The average change of striatal HVA concentration corresponds to a mean of 27.7% increase on the EDSC-injected side compared to the sham-injected side of the MPTP-treated monkeys (abbreviations: Sham: Sham-injected side of the MPTP-treated monkeys; EDSC: Endometrium-derived stem cell injected side of the MPTP-treated monkeys).

To assess the effectiveness of stem cell transplant as a potential treatment for PD, the dopamine metabolite HVA was measured in the brains of each animal. The intracranial transplant of endometrium-derived stem cells in MPTP-treated monkeys was associated with biochemical differences between the sides. Striatal HVA concentrations were higher in the EDSC-transplanted side of the brain in all three recipient monkeys. EDSC injection resulted in an average of an 8.2 ng/ml (27.7%) increase in HVA concentrations on the recipient side of the brain compared to the sham-injected side (*t*-test, *P* = 0.03; Fig. 4).

## Discussion

In this non-human primate study, we demonstrate the feasibility of allogeneic transplantation of endometrial stem cells to the striatum of asymptomatic MPTP-treated monkeys. We also show cell survival in the striatum, where they are able to incorporate, differentiate into a small number of tyrosine hydroxylase positive cells and improve striatal dopamine production.

In our previous study using a mouse model, we showed the ability of human endometrium-derived stem cells to differentiate into dopamine-producing, neuron-like cells *in vitro*, as demonstrated by their characteristic neuronal morphology, the presence of a barium-sensitive G-protein-coupled inwardly rectifying potassium current and tyrosine hydroxylase expression [7]. We also demonstrated that the transplantation of human endometrium-derived stem cells results in greater striatal dopamine levels in the MPTP-treated mouse model of PD.

Cell-replacement therapies in PD have focused on implanting stem cells that would protect the existing neurons or the neurons derived from other stem cell treatments, as well as transplanting stem cell-derived DA neuron precursors into the striatum [17]. Human foetal mesencephalon-derived cells, ES or iPS derived neuroblasts and subventricular zone adult neural stem cells have been used to derive DA neuron precursors [18–22]. However, human foetal mesencephalon cells and ES are not readily obtainable. DA neuron precursors derived from iPS or ES can form tumours after transplantation, unless they are entirely purified. Additionally, evidence suggests that even animal models using these cells cannot predict the risk of tumour formation in humans, which raises concerns about patient safety. Even a minor risk of tumour formation is unacceptable in the management of neurodegenerative diseases like PD because, unlike rapidly progressive neurodegenerative disorders, these diseases have a life expectancy near to that of the general population and have symptom-relieving treatment that can be administered even in advanced stages of disease. Moreover, patient derived iPS cells and their DA producing progeny may exhibit a gene profile that would make the patient specific DA cells susceptible to PD pathology and disease recurrence [17]. Altogether, the ideal stem cell type should be easily accessible, low risk, genetically resistant to disease, and immunologically acceptable for the patient (*i.e*. an autologous stem cell source).

Alternatively, endometrium-derived stem cells, a type of mesenchymal stem cell, are accessible with minimal or no analgesic requirements during a simple and routine office procedure. Unlike ES, their use is not limited by ethical concerns. Once collected, they can be expanded in large quantities *in vitro* and used for autogenic or allogeneic stem cell transplantation with no risk of teratoma. Furthermore, many Phase I/II clinical trials have documented the biosafety of MSC therapies [23]. Additionally, MSCs show remarkable regeneration capacity and can differentiate to dopamine-producing cells, insulin producing cells, urothelial cells, megakaryocyte-like cells, chondrocytes and cardiomyocyte-like cells [7,24–61].

Since our publication of the first evidence for neurogenic differentiation of endometrial stem cells, multiple groups have evaluated their neurogenic potential [29,30,34,41–43]. Consistent with our observations in the murine model of PD, the transplanted EDSCs can migrate and engraft to the injured area [7]. Previous research has shown that after engraftment, MSCs are somewhat protected from immune responses through their immunomodulatory effects, which thereby increase the long-term success of transplantation [5,62]. After homing to the site of injury, they release neurotrophic factors such as Neuronal Growth Factor, Brain Derived Neurotrophic Factor and Glial Derived Neurotrophic Factor, which hasten endogenous repair [4,23,63,64]. Additionally, evidence suggests that MSCs can be genetically modified and used as vectors to enhance the secretion of neurotrophic factors [65]. In addition to secreting neurotrophic factors, MSCs have been shown to regulate inflammation, decrease neuronal apoptosis, enhance myelinization, reconstruct neuronal circuitry, and promote endogenous neuronal regeneration [4].

As a result of their immunomodulatory effects, MSCs may be transplanted along with DA precursors derived from other stem cell types to increase their survival after transplantation. In parallel with these studies, we observed substantially more TH(+) cells in the transplanted side of the brain. Low sample size (*n* = 3) may explain why the difference between the cell counts did not reach statistical significance in this pilot feasibility study. We have also demonstrated that the transplanted cells recapitulated normal cellular allocation, exhibited long neurite-like projections, and expressed the rate-limiting enzyme of dopamine synthesis: tyrosine hydroxylase. The exact mechanisms of increased neuronal protection and HVA concentrations after endometrial derived stem cell transplantation need to be studied further. However*,* our preliminary data suggests that it is a combination of the neuroprotective effect of the EDSCs and their active contribution to dopamine production. Therefore, EDSC transplantation seems to ameliorate the two main pathophysiological mechanisms in PD. Stem cell therapies may provide enough therapeutic benefit for some patients that common drug treatments, such as L-DOPA, could be discontinued for several years [66]. Additionally, early decision-making models have shown the cost-effectiveness of stem cell therapies for early onset PD patients with debilitating symptoms [67]. These promising results suggest that stem cell therapies may become clinically competitive to existing therapies in the future, provided that an optimal source of DA neurons is discovered.

In conclusion, the endometrium represents an important source of stem cells with remarkable regeneration and differentiation capacity. After intracerebral injection, EDSCs can engraft striatum, migrate to the foci of cellular injury, differentiate to TH(+)neuron-like cells, and protect endogenous dopaminergic neurons, which ultimately results in higher dopamine concentrations. Although stem cell therapy for PD is in its relative infancy and a reliable source of DA cells needs to be determined, our preliminary primate study suggests that EDSCs deserve more extensive and detailed study for therapeutic potential in future clinical applications.
